# Impact of healthcare reform on the payer mix among young adult emergency department utilizers across the United States (2005–2015)

**DOI:** 10.1097/MD.0000000000013556

**Published:** 2018-12-10

**Authors:** Haley Bush, Lynn H. Gerber, Maria Stepanova, Carey Escheik, Zobair M. Younossi

**Affiliations:** a Betty and Guy Beatty Center for Integrated Research, Inova Health System; b Center For Liver Disease, Department of Medicine, Inova Fairfax Hospital, Falls Church, VA, United States.

**Keywords:** emergency department, health policy, health reform, insurance type, young adults

## Abstract

Before the patient protection and affordable care act (ACA), young adults (20 to 34) had the highest uninsured rates in the United States (US) and frequently sought care in emergency departments (EDs).

We aimed to determine if there was a measurable effect of expanded coverage, specifically the dependent coverage provision and Medicaid expansion, on the payer mix of young adults in EDs.

We performed a retrospective cross-sectional study of ED utilization among young adults across the US using the national hospital ambulatory medical care survey (NHAMCS) (2005–2015).

We examined the effect of health reform changes on the prevalence and odds of having an insurance type among ED utilizers (19–30) in 3 time periods (2005–2010), (2011–2013), and (2014–2015). Additionally, we compared the national and ED payer mix proportions among 19 to 25 and 26 to 30-year-olds.

Our results indicate significant proportional changes in the national and ED payer mix relative to a pre-ACA time period. The 2 greatest changes to the national payer mix were the reduction in the proportion of uninsured/self-payers and the increase in the proportion covered by Medicaid. Furthermore, the dependent coverage provision was effective in increasing the proportion of those (19–25) utilizing private insurance coverage. Lastly, there is now a lower proportion of uninsured young adults in the ED, and an increased proportion of those covered by Medicaid.

The change in payer mix among young adults has potential long-term consequences for the provision of emergency department services in the U.S.

## 1 Introduction

In 2010, young adults ages 20 to 34 comprised nearly 20% (62.6 million) of the United States population, but almost 30% did not have health insurance.^[1,2]^ That same year, an estimated 49.9 million Americans, 16.3% of the population, did not have health insurance.^[3]^ Consequently, young adults (20–34) had the highest uninsured rates of all age groups encompassing approximately 40% of all uninsured Americans.^[2]^ Several reasons have been suggested to explain why young adults are over-represented in the uninsured group; limited income, a lack of employer-based coverage, lack of affordable coverage through postsecondary education, or restricted access to healthcare.^[4–8]^

In 1986, Congress enacted the emergency medical treatment and labor act (EMTALA) to ensure public access to emergency services regardless of ability to pay.^[9]^ This federal law results in emergency departments (EDs) serving as the “safety net of the safety net” for uninsured patients and Medicaid beneficiaries.^[10,11]^ Consequently, numerous studies have established that young adults utilize ED care for their health related concerns.^[12–14]^ One study determined that ED care accounts for 21.6% of all healthcare visits from adults 20 to 29, and that between 2005 and 2010, there was a 15% increase in ED visits in California among those 19 to 34.^[12–14]^ The passage of the EMTALA in conjunction with our changing healthcare system has contributed to the growing volume of ED visits, particularly among young adults.^[11–14]^

The patient protection and affordable care act (ACA) was signed into law on March 23, 2010, with a primary goal of increasing the number of Americans with health insurance.^[15]^ The dependent coverage provision, enacted on September 23, 2010, allowed for adults 19 to 25 in all states to obtain coverage under a parent's employer-sponsored or individually purchased health insurance plan.^[15]^ Furthermore, the ACA allowed for the expansion of Medicaid, beginning January 1, 2014, which increased coverage to include adults with income up to 133% of the federal poverty level (FPL).^[15]^ Twenty-six states signed legislation that expanded Medicaid effective January 1, 2014.^[16]^

According to the Department of Health and Human Services, over 6.1 million adults 19 to 25 gained health insurance coverage through the ACA.^[17]^ An estimated 2.3 million gained coverage as a result of the dependent coverage provision and 3.8 million gained health insurance coverage from the Medicaid expansions and the individual exchanges as of early 2016.^[17]^ While it is clear that these provisions have had a beneficial impact on increasing the number of young adults with health insurance, there is a lack of clarity regarding how this increase in coverage has affected ED utilization by insurance status. Several studies have examined the impact of the dependent coverage provision and Medicaid expansion on ED visits and volumes, but the findings have not been consistent. Two studies concluded that the dependent coverage provision decreased ED use and visits,^[18,19]^ while Medford-Davis et al determined there were significant increases in ED volumes.^[20]^ Furthermore, 1 study determined that ED visits increased after a state expanded Medicaid, while a review of 28 states plus the District of Columbia determined there was no statistical significant increase in ED volumes.^[21,22]^

In this study we examine how the national payer mix and ED payer mix have changed in a post-ACA period relative to a pre-ACA period. Specifically, we compare differences between the 19 to 25-year-olds in the U.S to 26 to 30-year-olds who were no longer covered by parental insurance. We aim to determine if the proportionality of certain insurance types increased or decreased during the selected time periods. The study inspects 1 pre-ACA time frame (2005–2010) and 2 post-ACA time frames (2011–2013) and (2014–2015). The division of the study into these time frames aims to assess how different ACA provisions differentially impact the coverage distribution of 19 to 25-year-old ED utilizers relative to an older age group, 26 to 30. These divisions permitted us to examine the potential impact of 2 important ACA provisions: the dependent coverage provision and Medicaid expansion. We assessed the proportional changes in the ED payer mix during the post-ACA periods relative to the pre-ACA time frame.

## 2 Methods

### 2.1 Data source

For this study, we used the national hospital ambulatory medical care survey (NHAMCS).^[23]^ This survey was originally designed to collect data on the utilization and provision of ambulatory care in hospital emergency rooms. It is based on a national sample of visits to those departments in non-institutional general and short-stay hospitals (exclusive of Federal, military, and Veterans Administration hospitals). The data are publicly available to download from the National Bureau of Economic Research website.^[23]^

Sampling design of the NHAMCS dataset utilizes a 4-stage probability sampling. It starts with sampling of geographically defined areas, followed by sampling of hospitals within these areas, followed by sampling of clinics within outpatient departments (all emergency service areas and in-scope ambulatory surgery locations are included), and then of individual patient visits within sampled departments. In the resulting sample, 1 record represents 1 visit.^[23]^ For this study, we used NHAMCS Emergency Department data for the years 2005 through 2015.

### 2.2 Study sample and definitions

For the purpose of this study, we selected people between 19 and 30 years of age. They were further sub-divided into 2 groups: 19 to 25 and 26 to 30. Furthermore, the study years were merged into 3 different time periods: 2005 to 2010 (pre-ACA), 2011 to 2013 (dependent coverage provision), and 2014 to 2015 (Medicaid expansion). The significance of the age and time categorization was to assess the impact of varying provisions of the ACA on insurance coverage for ED utilizers. The age categorization was utilized to identify how the dependent coverage provision impacted those 19 to 25 relative to those 26 to 30, which are a relatively similar group of individuals. Each ED visit collected data on the primary payer divided into the following insurance types: private insurance, Medicare, Medicaid, worker's compensation, self-pay, no charge/charity care, or other/unknown.

### 2.3 Statistical analysis

Ultimate stratum and cluster units provided with the data were used to account for the survey design, and individual visit weights were used to produce national estimates. There were 41,553 emergency department visits in the 19 to 25-year-old age group that were included in the dataset (2005–2015), merged into 1604 clusters and 62 stratum units. The prevalence rates of all insurance types were calculated for each age group in each study period, and were compared between age groups and across study periods using Rao-Scott Chi-square Test. The same test (for categorical) or a linear regression model (for continuous) was used to compare other collected parameters between those groups. Multinomial logistic regression models with adjustment for patients’ demographics and parameters of the visit were used to evaluate independent association of a study period with having private insurance or Medicaid (reference: uninsured), without and then with interaction of predictors with the age group. *P*-values of.05 or less were considered statistically significant and are indicated by an asterisk (^∗^) in the tables. The study was reviewed and approved by the Inova Institutional Review Board. All analyses were run using SAS 9.4 (SAS Institute, Cary, NC).

## 3 Results

### 3.1 Differences in insurance coverage among 19 to 25-year-old emergency department utilizers relative to the national prevalence of insurance holders

Table 1 represents a comparison between the national and ED prevalence of insurance types. The national data were published by the National Health Interview Survey (NHIS) (24) and the ED data were gathered from our analysis of the NHAMCS. The NHIS categorized insurance into 3 groups: private, public, and uninsured.^[24]^ However, the NHAMCS used more specific insurance categorizations, described in the methods section above. Therefore, those with Medicare and Medicaid were combined for the public insurance group and the self-payers and no charge groups into the uninsured category. Those with private insurance were classified as the private coverage group. Consequently, 2 groups remained unclassified in the ED data for comparison to the national data: worker's compensation and unknown. The comparison between the national and ED data is meaningful as the combination of worker's compensation and the unknown category remained relatively constant over the study years (12.5% to 16.8%) (Table 1).

**Table 1:**
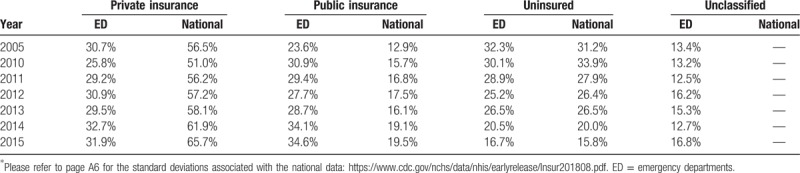
Distribution of insurance types from (2005–2015) among emergency department utilizers versus the overall national estimations among 19–25-year-olds.

The national prevalence of private insurance, public insurance, and no insurance in 2005 (56.5%, 12.9%, and 31.2%) transformed to (65.7%, 19.5%, 15.8%) in 2015 (Table 1), demonstrating a notable drop in the percentage of uninsured in this age group. The ED distribution of private coverage, public coverage, and no insurance in 2005 (30.7%, 23.9%, and 32.3%) changed to (31.9%, 34.6%, and 16.7%) in 2015 (Table 1), demonstrating a significant drop in the ED usage by uninsured in this age group, consistent with the national decline of uninsured in this age group.

### 3.2 Prevalence of insurance types among 19 to 25 and 26 to 30-year-olds emergency department utilizers over time

Table 2 depicts the ED payer mix distribution among those 19 to 25 and 26 to 30 over the 3 specified time periods. The table is divided into insurance type, with the exclusion of Medicare and workers’ compensation (because of the small populations in these types for the specified age groups), and follows the changes in proportion of coverage types over time. Within each age group, significant changes in payer mix were assessed by comparing 2 time periods (2005–2010 to 2011–2013; 2011–2013 to 2014 and 2014 to 2015).

**Table 2:**
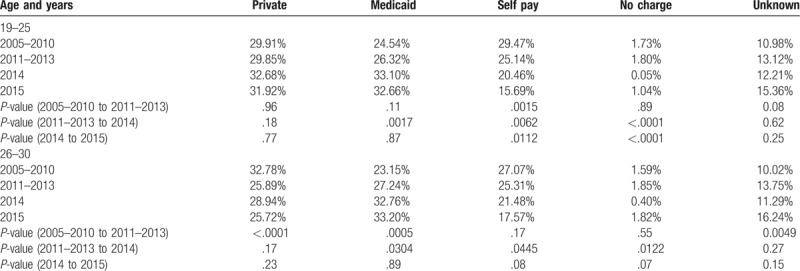
Prevalence of insurance type by year among emergency department utilizers.

From the (2005–2010) to (2011–2013) period, ED utilizers 26 to 30 experienced a significant decrease in the proportion of private payers (*P* < .0001), while those 19 to 25 remained unchanged (*P* = .96) (Table 2). At the same time, ED utilizers 26 to 30 experienced significant proportional increases in Medicaid coverage from the (2005–2010) to (2011–2013) period (*P* = .0005) and from the (2011–2013) to 2014 period (*P* = .03), as did those 19 to 25 (*P* = 0.0017) (Table 2). Furthermore, 19 to 25 ED utilizers experienced significant proportional decreases in the self-payer group from the pre-ACA to (2011–2013) period (*P* = .0015), from the ( 2011–2013) to 2014 (*P* = .0062), and then to 2015 (*P* = .0112) (Table 2).

Table 3 illustrates significance differences in the prevalence of ED payer mix between the 2 age groups 19 to 25 and 26 to 30 within the 3 specified time periods. Table 3 is in direct support of Table 2 as it identifies significant differences in the proportion of different types of coverage data between the 2 age groups presented in Table 2.

**Table 3:**
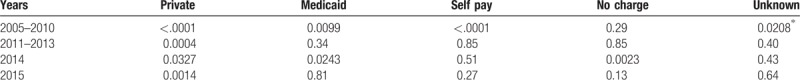
Significance comparison of insurance type by age (19–25 vs. 26–30) among emergency department utilizers.

### 3.3 Odds of being covered by a specific insurance type for an ED visit in 2014 and 2015 relative to a Pre-ACA time period (2005–2010)

A multinomial logistic regression analysis, adjusted for gender, race, and region was conducted to examine the odds of having a specific insurance type among ED utilizers in 2014 and 2015 relative to the pre-ACA time frame (2005–2010) by age category.

The odds of being privately insured for the 19 to 25 age group was 1.69 in 2014 and 2.13 in 2015 when compared to the 2005 to 2010 period (both *P* < .001). The same odds were 1.12 in 2014 and 1.26 in 2015 for the 26 to 30 age group (both *P* > .10); those were significantly lower when compared to the respective odds for the 19 to 25 group cited above (both *P* < .0006) (Table 4).

**Table 4:**
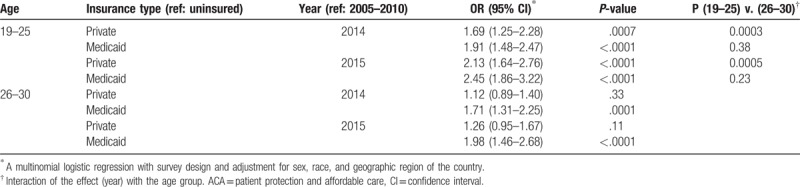
Odds of emergency department utilizers having a specific insurance type in 2014 and 2015 versus the pre-ACA time period (2005–2010).

On the other hand, the odds of being covered by Medicaid among ED utilizers, over the same time period, indicated significant increases within each age category, and there was no statistically significant difference between the 2 age groups (all *P* > .20). The odds of being covered by Medicaid for an ED visit among those 19 to 25 was 1.91 in 2014 and 2.45 in 2015 (both *P* < .0001) while it was 1.71 in 2014 and 1.98 in 2015 (both *P *≤.* *0001) among 26 to 30-year-olds (Table 4).

## 4 Discussion

From 2010 to 2015, among 19 to 25-year-olds there has been a national increase from 51% to 65.7% of those covered by private insurance, an increase from 15.7% to 19.5% of those covered by public insurance, and a decrease from 33.9% to 15.8% of those uninsured.^[24]^ The most notable changes were the increase in private insurance and decrease in the uninsured. The Commonwealth Fund estimated that nearly 13.7 million adults 19 to 25 stayed on or joined their parents’ health plans in the beginning of the 2011.^[25]^ Numerous other studies assessing the impact of the dependent coverage provision have established that the policy significantly increased private health insurance coverage for those 19 to 25.^[26–29]^ These findings support the visible growth trends among the proportion of those (19–25) privately insured in the ED from 2010 to 2014 (25.8% to 31.9%). Furthermore, McMorrow et al examined the national impact of the Medicaid expansion on young adult coverage and found an increase in public coverage to 21.8% in 2014 and a drop in uninsurance to 19.6%.^[30]^

Our study findings identified a difference in payer mix between the national and the ED insurance coverage distribution. This proportional difference in payer mix existed during the pre-ACA time period and persisted into the post-ACA time periods. Before the ACA was signed into law, over half of 19 to 25-year-olds were covered by private insurance, 13% by public insurance, and 31% were uninsured.^[24]^ During this same time period, private insurance covered 31%, public insurance covered 24%, and the uninsured represented 32% of all ED visits made by those 19 to 25. These numbers indicate a larger proportion of public insured users, a smaller proportion of privately insured users, and a comparable proportion of uninsured users in the ED relative to the national breakdown. These findings are supported by Zuckerman et al who concluded that the uninsured do not have more ED visits than the insured population. Instead, the publicly insured are overrepresented among ED users, likely because this population needs more overall care.^[31]^ Furthermore, Garcia et al determined that Medicaid beneficiaries were more likely to have had a least 1 ED visit in a 12-month period than persons with private insurance and the uninsured.^[32]^

By 2014, the coverage for dependent young adults had been established policy for several years and the Medicaid expansion was just taking effect. The national payer mix and ED payer mix changed relative to the pre-ACA period. By 2015, 65% of those 19 to 25 were covered by private insurance, 19% by public insurance and the uninsured had fallen to 16%.^[24]^ These national payer changes were also reflected in the ED utilization. Private insurance was covering 32% of ED visits, public insurance 35% of visits, and uninsured 17% of visits. It is clear that the passage of the ACA did not mitigate the proportional difference in payer mix between the national and ED insurance coverage distribution. In fact, the proportional differences that existed before the ACA were further increased for those covered by private and public insurance. Several other studies have examined the payer mix of ED utilizers after the expansion of Medicaid on the national and individual state levels. In 2008, a health insurance experiment conducted in Oregon demonstrated that expanding Medicaid had the causal effect of increasing emergency department use among those covered by Medicaid.^[33]^ Furthermore, Tang et al examined trends in ED visits across the US and found that ED visit rates among those with Medicaid increased significantly, while those with private insurance or uninsured did not.^[11]^ Tang partially attributed this finding to the massive increase in Medicaid enrollment (4.8 million) over the study period.^[11]^ Lastly, the Medicaid expansion in California was associated with increases in ED visits paid for by Medicaid and decreased in uninsured visits.^[34]^ The proportional difference in the ED payer mix will likely have affects for both patients and ED providers as evidence suggests that those covered by Medicaid are charged the most for ED visits, followed by private insurance, and then uninsured.^[35]^ The growing proportion of young adults covered by Medicaid combined with the decreasing proportion of the uninsured will have impacts on the economics of ED care.

The ACA differentially impacted the insurance coverage for those 19 to 25 and 26 to 30 ED utilizers over the 3 different time periods. Before the passage of the ACA, the 26 to 30 group had significantly more private insurance coverage in EDs relative to the 19 to 25 group, who were significantly more likely to be covered by Medicaid or to be uninsured. During this time, those 19 to 25 had less access to private insurance because this group was often dropped from their parents’ private policies or public insurance programs at the age of 19.^[36]^ Likewise, jobs for young adults generally pay lower wages or are temporary and not supplemented with health benefits,^[36]^ and 2005 to 2010 was a period of recession and relatively little employment expansion. For these reasons, the dependent coverage provision was written as a provision of the ACA in an attempt to bridge the gap of access to health insurance for those 19 to 25. Not only did the dependent coverage provision increase the national proportion of those covered by private insurance, it impacted the proportion of those visiting the ED. In particular, comparing the proportion of 19 to 25 ED utilizers with private insurance in 2010 (25.8%) to 2011 (29.2%) demonstrates the dramatic immediate impact of the dependent coverage provision. Furthermore, the dependent coverage provision was extremely effective in increasing the odds of those 19 to 25 being covered by private insurance for ED visits relative to the 26 to 30 group. Compared to the pre-ACA time frame, in 2015, the odds of those 19 to 25 being covered by private insurance for an ED visit was 2.13 (*P* < .0001) which was also statistically significantly different from the 1.26 for those 26 to 30 (*P* = .0003). Our data do show that private insurance coverage for the 26 to 30-year-olds did drop significantly from the period 2005 to 2010 compared with 2011 to 2013, but not from the latter period compared with 2014 or from 2014 to 2015. Several other studies found a relative increase in the probability of private insurance coverage and a decrease in uninsured among those 19 to 25.^[18,37]^

In contrast to changes in private insurance coverage, the Medicaid expansion equally improved the odds of those 19 to 25 (2.45) and 26 to 30 (1.98) being covered by Medicaid for ED visits in 2015 relative to the pre-ACA time period (*P* = .23 between the 2 age groups). During 2014, the proportion of those uninsured 19 to 25 and 26 to 30 decreased with significance relative to the (2011–2013) time period. In 2015, the proportion of uninsured among both age groups fell to below 18% of all ED visits and were not statistically significantly different from one another. Pines et al confirms that Medicaid-paid ED visits increased in expansion states staring in January 2014 and uninsured visits decreased, likely because uninsured individuals gained Medicaid coverage.^[22]^ Further support for our findings come from Kelin et al examining the effect of Medicaid expansion on ED utilization in Maryland and concluding that there was a substantial increase in patients covered by Medicaid in the post-ACA time period.^[38]^

While this study has many strengths, it should be interpreted in light of several limitations. First and foremost, this study used a non-randomized observational design and thus cannot prove that specific ACA provisions caused changes in ED utilization reported in this study. However, examining the trends and changes over an expanded period of time helps mitigate this limitation. Secondly, this study is nationally representative and does not take into account the state-by-state variations in pre-existing laws. Before the passage of the ACA, several states had laws extending private coverage to young adults and several had expanded Medicaid coverage. Furthermore, only 26 states expanded Medicaid beginning in 2014. It is therefore important to realize that the study conclusions pertain to national trends only and not state specific changes. Thirdly, the dependent coverage provision began to take effect on September 23, 2010. However, the insurance plans were formally enacted when the contracts were renewed. The renewal of insurance contracts could have occurred any time up until September 23, 2011. For this reason, we began examining the data beginning on January 1, 2011 and averaged the results over a 3 year time period (2011–2013) to gain a more accurate representation of the true effect of the provision. Our study observes the effects of Medicaid expansion from 2014 to 2015. While we obtained significant results, these are only preliminary data, and will need to be explored in future research. Lastly, the data are presented as proportions of the total people covered and visiting ED. We do not have access to the actual numbers of people accessing ED care, an important piece of data if one is to assess whether health care legislation in fact influences how many people access ED care.

In conclusion, we explored changes in insurance coverage for 2 different age groups over a period of significant change in health care legislation. The data demonstrate that there has been a national reduction in the uninsured rates of young adults. This trend was reflected in data from ED utilization as well. There are proportional differences in the national insurance coverage prevalence relative to the ED payer mix that have persisted into the post-ACA time frame. There is a larger proportion of public coverage, a smaller proportion of private and equal number of uninsured ED users relative to the national coverage prevalence. Furthermore, there are established differences in the ED payer mix between adults 19 to 25 and 26 to 30 throughout the study. Our findings indicate that the dependent coverage provision was effective in increasing the proportion of those 19 to 25 utilizing private insurance coverage from the passage of the ACA to 2015. We did not see a significant increase in private insurance coverage for 26 to 30-year-olds.

Our data support the conclusion that there are now a lower proportion of uninsured young adults in the ED, thereby accomplishing one of the primary goals of the ACA, to reduce the uninsured. The data also demonstrate that this reduced proportion of uninsured young adults has resulted in an increased proportion of those covered by private insurance and Medicaid. If the trend of increasing proportional representation of Medicaid users in the ED continues, there are potential long-term consequences for the provision of emergency department services in the U.S.

## Author contributions

**Conceptualization:** Haley Bush.

**Data curation:** Maria Stepanova.

**Formal analysis:** Maria Stepanova.

**Investigation:** Haley Bush.

**Methodology:** Maria Stepanova, Zobair Younossi.

**Project administration:** Carey Escheik.

**Resources:** Zobair Younossi.

**Supervision:** Lynn Gerber, Zobair Younossi.

**Visualization:** Lynn Gerber.

**Writing – original draft:** Haley Bush, Zobair Younossi.

**Writing – review & editing:** Haley Bush, Lynn Gerber, Maria Stepanova, Carey Escheik, Zobair Younossi.

Haley Bush orcid: 0000-0003-0361-5150.
